# Purification strategy and effect of impurities on corrosivity of dehydrated carnallite for thermal solar applications

**DOI:** 10.1039/c9ra09352d

**Published:** 2019-12-16

**Authors:** Youyang Zhao, Noah Klammer, Judith Vidal

**Affiliations:** National Renewable Energy Laboratory 15013 Denver West Parkway Golden CO 80401 USA Youyang.zhao@nrel.gov

## Abstract

This paper presents a purification method for dehydrated carnallite (DC)—a commercial ternary MgCl_2_–KCl–NaCl salt—for concentrating solar power (CSP) applications based on a thermal and chemical treatment using the reduction power of Mg. The purification is effective at reducing MgOH^+^ by an order of magnitude—from around 5 wt% in non-treated salt to less than 0.5 wt% in post-purification salt. The corresponding decrease in the measured corrosion rate of Haynes 230 at 800 °C from >3200 μm per year to around 40 μm per year indicates that soluble MgOH^+^ is indeed correlated to corrosion. The addition of elemental Mg serves as both a scavenger of impurities and corrosion potential control, which are considered the primary mechanisms for corrosion mitigation.

## Abstract 2

Thermal and chemical purifications empower molten chlorides for next generation concentrating solar power (CSP) applications.

## Graphical abstract

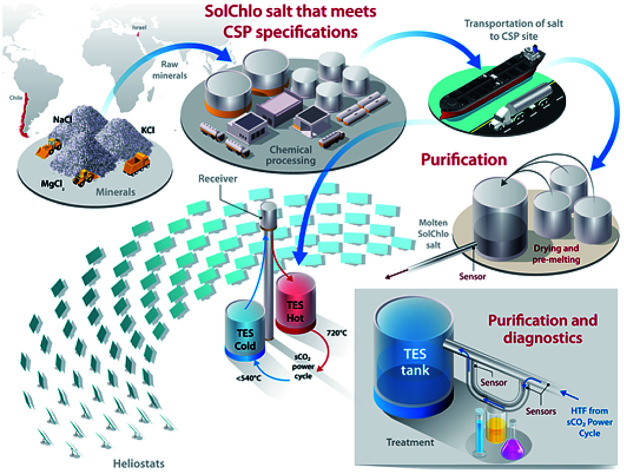

## Introduction

To enable the use of a supercritical carbon dioxide (sCO_2_) Brayton power cycle, next-generation (Gen3) concentrating solar power (CSP) requires a heat-transfer fluid (HTF) and thermal energy storage (TES) medium that can operate in the temperature range of 500–750 °C. This operational temperature differs from the current Gen2's molten nitrate HTF and TES medium, which is stable up to ∼565 °C. Gen3 CSP's higher operating temperature range demands new chemistry with high thermal stability and low corrosion on metallic materials used in the receiver, primary heat exchangers, piping, and TES tanks.

Dehydrated carnallite (DC)—a commercial ternary MgCl_2_–KCl–NaCl chloride-salt—has been proposed given its thermal stability up to ∼800 °C, low cost, and availability. The liquidus temperature of the DC salt is also around 440–450 °C, which also falls into the operating temperature range of the sCO_2_ Brayton cycle. However, the corrosion behavior of the ternary salt is less known. The hygroscopic nature of the MgCl_2_ component complicates the investigation because dehydration of the MgCl_2_-containing salt is not as simple as heating up the mixture to different temperatures to remove different forms of MgCl_2_ hydrates. The hygroscopic nature of MgCl_2_ has been known for a long time in the literature.^1–9^ Stepwise dehydration of MgCl_2_·6H_2_O to form lower hydrates can be achieved by heating MgCl_2_·6H_2_O to different temperatures as shown by eqn (1a)–(1d).^4^

1a MgCl_2_·6H_2_O = MgCl_2_·4H_2_O + 2H_2_O at ∼117 °C

1b MgCl_2_·4H_2_O = MgCl_2_·2H_2_O + 2H_2_O at ∼180 °C

1c MgCl_2_·2H_2_O = MgCl_2_·H_2_O + H_2_O at ∼240 °C

1d MgCl_2_·H_2_O = MgCl_2_ + H_2_O at ∼400 °C

However, hydrolysis reactions during dehydration to form MgOHCl and HCl, as shown in eqn (2a) and (2b), occur simultaneously in a similar temperature range (of >240 °C) as dehydration:^3–5,8,10,11^

2a MgCl_2_·2H_2_O = MgOHCl + HCl(g) + H_2_O

2b MgCl_2_ + H_2_O = MgOHCl + HCl(g).

It is very challenging to control the formation of MgOHCl during dehydration in a normal atmosphere. Dry HCl gas with partial pressure exceeding values calculated by Kipouros and Sadoway^4^ can inhibit the hydrolysis reactions based on Le Chatelier's principle. During production of the chloride salt electrolyte for Mg production at Israel Chemicals Ltd (ICL), Cl_2_ gas is bubbled through the molten DC in a process used by ICL for controlled dehydration. At high temperatures of 533–555 °C, MgOHCl thermally decomposes to form MgO and HCl gas as shown in eqn (3).^4,12^

3 MgOHCl = MgO + HCl(g)

From eqn (2) and (3), both the formation and thermal decomposition of MgOHCl lead to formation of highly corrosive HCl gas. Combined with the H_2_O released during dehydration, HCl can be detrimental to most alloy components. In addition, dissolved MgOHCl in molten chlorides, in the form of MgOH^+^, is also known to corrode alloys, as given by eqn (4a) and (4b),^13^ where M can be a pure metal or a metal component in an alloy such as Fe, Cr, or Mn:

4a MgOHCl = MgOH^+^ + Cl^−^

4b *x*MgOH^+^ + M = *x*MgO + M^*x*+^ + (*x*/2)H_2_

Therefore, an effective method is needed to remove MgOHCl from the chloride salt before performing corrosion.

By leveraging the experience from the magnesium production industry, which has been using similar MgCl_2_-containing salt for decades, and from past research in the literature,^4,14^ we designed a thermal and chemical purification procedure that is effective at removing MgOHCl and other cationic impurities in the commercial salt. 100 hour corrosion evaluation at 800 °C was performed on Haynes 230, which is of interest to Gen3 CSP technology to serve as the solar-receiver material because of its high temperature stability and chemical resistance. DC salts subjected to different purifications were used for corrosion evaluation while their pre-corrosion MgOHCl content was tracked by an analytical titration technique.^15^ The objectives of our study are to (1) understand the corrosiveness of the ternary chloride salt, (2) investigate the effectiveness of each purification process, (3) verify that MgOHCl is indeed correlated to corrosion, and (4) design future corrosion mitigation strategies.

## Materials and methods

### Materials

The salt used for corrosion was “dehydrated” carnallite supplied by ICL. The primary chemical constituents of DC are KCl and MgCl_2_ in a chemical formula of KMgCl_3_, whose mineralogical name is carnallite. There is an additional 5–7 wt% of NaCl in the DC based on the mineralogical resource from the Dead Sea where ICL is located. Table 1 shows the elemental composition of DC provided by ICL. Note that DC salt contains roughly 5 wt% water (in the form of chemical hydrate and/or physically absorbed moisture). Once received, the DC salt was stored in an MBraun glove box under nitrogen with <0.5 ppm H_2_O and <0.5 ppm O_2_.

**Table 1:** Carnallite elemental chemical analysis provided by ICL

K wt%	Mg wt%	Na wt%	Br wt%	Cl wt%	H_2_O wt%
21.2	12.8	1.33	0.58	58.2	5

### Salt purification

The dehydrated carnallite salt was purified to remove H_2_O and impurities before being used for corrosion with Haynes 230 coupons at 800 ± 10 °C for 100 hours. Salt purification consists of two separate parts: a thermal purification and a chemical purification. The thermal purification follows the principles of Kipouros and Sadoway,^4^ which provides the guideline for a stepwise dehydration process at 117 °C for 8 hours, 180 °C for 8 hours, 240 °C for 2 hours, 400 °C for 1 hour, and 600 °C for 1 hour, with a heating rate of 5 °C min^−1^ between the isothermal steps. The chemical purification follows the principles of using an active metal such as Mg or Zr to remove MgOHCl impurity as given by the German Aerospace Center (DLR) and Savannah River National Laboratory (SRNL).^13,16,17^ Following SRNL's suggestion based on their investigation, we used 1.7 wt% of elemental Mg chips (99.98% trace metals basis, 6–35 mesh, Sigma Aldrich). 1.7 wt% of Mg was a conservative estimation to ensure Mg is in excess. Investigation of a more accurate amount of Mg addition was determined in a follow-up study. After chemical purification, excess Mg, in the form of droplets at the bottom of the solidified salt, was removed.

### Corrosion setup

**Fig. 1:**
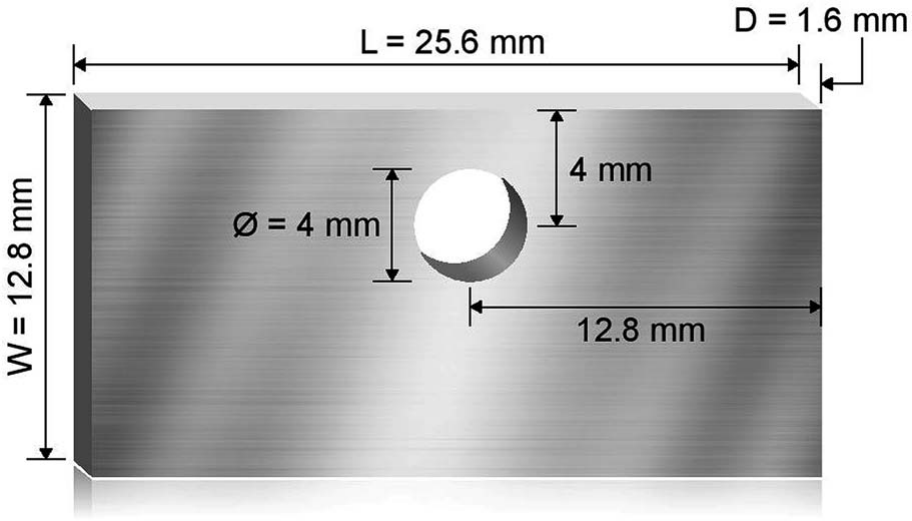
Schematic showing the dimension of the machined Haynes 230 coupons used for corrosion where *L* is the length, *W* is the width, and *D* is the thickness of the coupons. A hole of 4 mm diameter is cut to allow the coupon to be hung on a Ni wire.

**Fig. 2:**
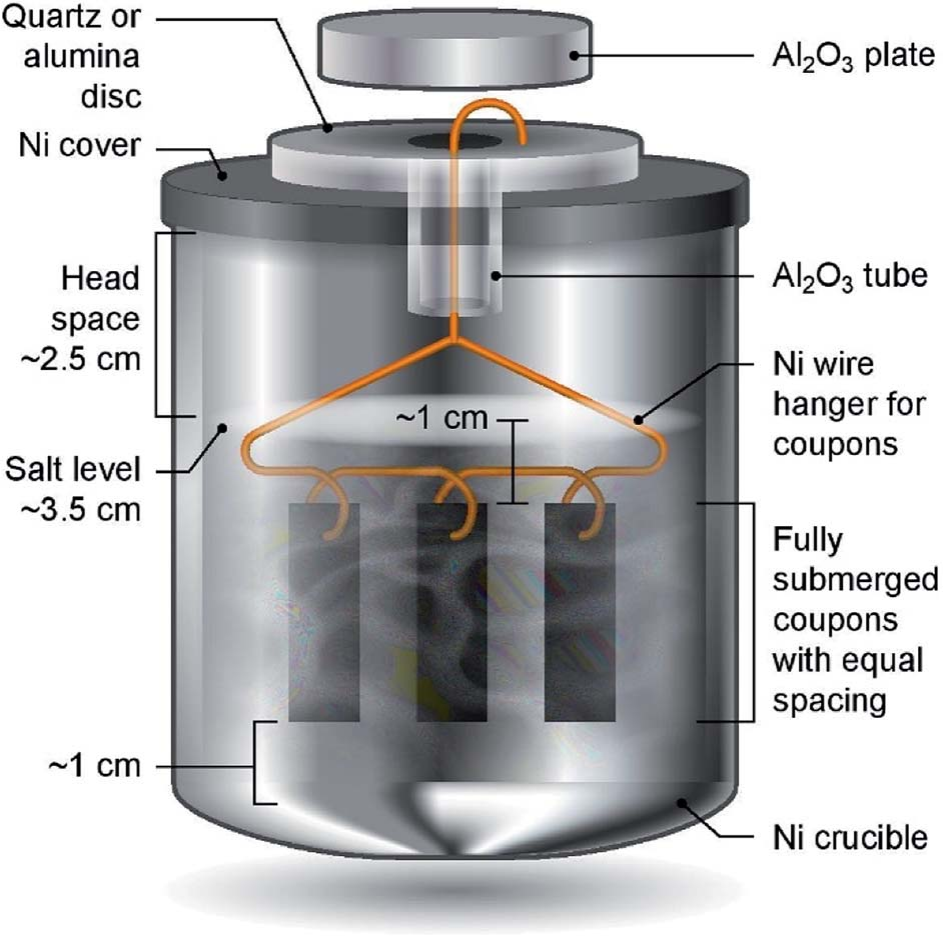
Schematic of the corrosion vessel setup (not to scale) showing all components and spatial arrangement of Haynes 230 coupons and salts for corrosion tests. Mg chips are blended into the salt mixture.

Haynes 230 supplied by Haynes International was cut by a waterjet cutter into coupons with dimensions shown in Fig. 1. The coupon surfaces were lightly polished with 120-grit sandpaper in water prior to corrosion tests. A 250 mL Ni crucible (Sigma-Aldrich Z246581) and a Ni crucible cover (Sigma-Aldrich Z245700) were used as the corrosion vessel. The crucible and cover were used as received without special treatment. Three Haynes 230 coupons were used in each corrosion test. A 4 mm-diameter hole was drilled at the center of the Ni crucible cover to form a mechanism to hang the Haynes coupons on a Ni wire (99.98%, 1 mm diameter, Goodfellow NI005171) into the molten chloride salt. The schematic in Fig. 2 shows the details of the corrosion vessel setup. We used a combination of alumina tube (3.08 mm outer diameter, 1.53 mm inner diameter, CoorsTek), alumina plate (2 mm thickness, CoorsTek), and quartz disc (25.4 mm diameter with a 3.2 mm-diameter center hole, AdValue Technology FQ-D-1N-N1/16) to prevent galvanic coupling between Haynes 230 coupons and the Ni crucible/cover. The spacing between Haynes 230 coupons was about 1 cm to allow ample space to minimize mass-transfer limitation during corrosion testing.

About 200 g of purified DC salt (after removal of excess Mg droplets) were placed into the Ni crucible. This amount of salt ensured full submersion of the Haynes 230 coupons in the molten salt during corrosion. The corrosion setup was then assembled according to Fig. 2. The assembly was next placed into a stainless-steel 309 (SS 309) bag (McMaster 3438K13). A piece of Ta foil (0.0127 mm thickness, certified pinhole-free, 99.95% trace metals basis, BeanTown Chemical) was inserted into the SS bag, on top of the assembly, serving as an oxygen getter. The opening of the SS bag was folded to avoid excess release of vapor from the corrosion vessel to the furnace test vessel during the corrosion test. The entire assembly was completed in the glove box under ultra-high-purity (UHP) nitrogen atmosphere and then transferred into the furnace test vessel within a few minutes. The furnace test vessel was sealed from ambient atmosphere with a grafoil gasket. The furnace test vessel was pumped down to −0.08 MPa and refilled with nitrogen gas (UHP, Airgas) at least three times to remove oxygen and moisture, after which UHP nitrogen gas was flowed continuously at 150 sccm during corrosion. The heating schedule shown in Table 2 was used for the corrosion.

**Table 2:** Heating schedule for the 100 hour corrosion test at 800 °C

Salt temperature range, °C	Ramp rate, °C min^−1^	Hold time, hour
25–117	5	8
117–800	5	100
800–25	Natural cooling	Not applicable

Post-corrosion sample preparation: after corrosion testing, the assembly was transferred to the glove box and the corroded Haynes 230 coupons were carefully retrieved after breaking the solidified salt. Next, the Haynes 230 coupons were taken out of the glove box, immersed in deionized (DI) water, and ultrasonicated for 15 min to remove residual salt. After ultrasonication, the coupons were rinsed with ethanol, dried, and stored in a desiccator. The coupons were cut by a diamond-blade saw about 6–7 mm from the short edge. The cut surface was mounted in phenolic resin and polished to a mirror finish for metallographic characterization with scanning electron microscopy (SEM) and energy-dispersive spectroscopy (EDS).

### Corrosion rate calculation

The corrosion rate *C*_1_ defined as the loss of metal surface thickness per unit time (*e.g.*, μm per year) is given by eqn (5) based on the initial metal-coupon surface area (*A*), the weight change of the metal coupons during corrosion test (Δ*W*), corrosion time (*t*), and metal density (*ρ*) assuming that uniform corrosion occurred on the Haynes 230 coupons and that there was no change of corrosion mechanism(s).

5 *C*_1_ = Δ*W*/*ρAt*

### MgOHCl content measurement

An analytical titration technique was developed at NREL.^15^ This titration method used the different solubilities and chemical reactions of chloride salt components, MgOHCl and MgO, in DI water and methanol to have successful physical separation of each species, followed by an ethylenediaminetetraacetic acid (EDTA) titration to determine Mg^2+^ content in each species. The method was able to detect MgOHCl content down to at least 0.1 wt% with standard deviation on the order of 0.01 wt% as reported by NREL.^15^

## Results

### MgOHCl content

**Fig. 3:**
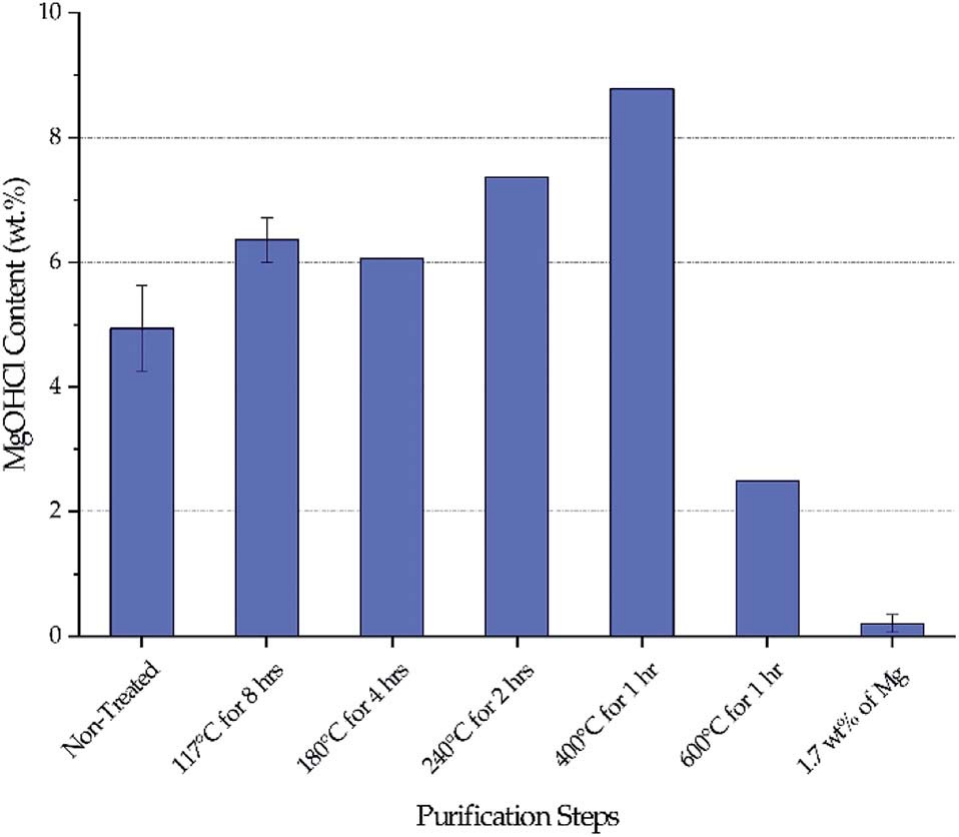
Variation of MgOHCl during thermal purification and chemical purification with 1.7 wt% of Mg chips. The same batch of salt was used to obtain data at different purification steps. Error bars are present when multiple measurements were available.

Fig. 3 shows the MgOHCl content measured by the analytical titration method. There is a gradual increase of MgOHCl during the first four isotherms of the thermal purification (*i.e.*, 117 °C, 180 °C, 240 °C, and 400 °C), followed by a sharp drop at the last isotherm of the thermal purification (*i.e.*, 600 °C). The MgOHCl content further drops during the chemical purification (*i.e.*, with 1.7 wt% of Mg). It should be noted that multiple measurements were performed on non-treated DC salt, DC salt after heat treatment at 117 °C for 8 hours, and thermally + chemically purified salt (*i.e.*, 1.7 wt% of Mg).

Our major objective is to detect the decrease of MgOHCl content by the thermal + chemical purification, so we focused our titration effort on the thermally/chemically purified salt sample (1.7 wt% of Mg in Fig. 3). We did not perform multiple titrations on every salt sample because the existing data suggested that the standard deviation of the titration measurements was at a fairly small level of less than 0.5 wt%. Nevertheless, we acknowledge that more measurements should have been performed to have standard deviation information. Hence, only qualitative (instead of quantitative) analysis of the data is given in the following Discussion section.

### Corrosion rates

**Fig. 4:**
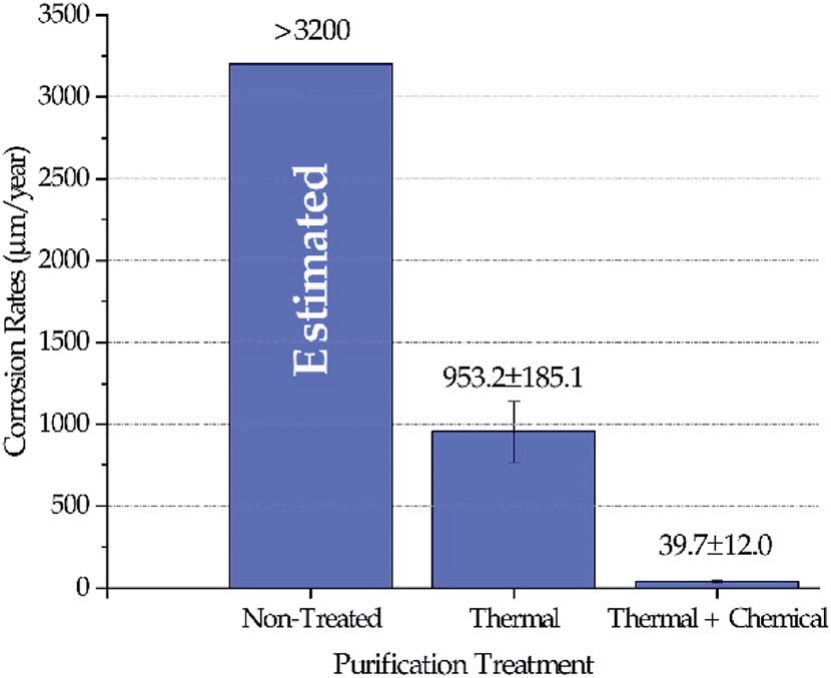
Corrosion rates of Haynes 230 coupons in molten DC salts at 800 ± 10 °C for 100 hours following different purification treatments. Corrosion rate in non-treated DC salt was estimated at 3200 μm per year or above.

Fig. 4 shows the corrosion rates of Haynes 230 coupons in molten DC salts at 800 ± 10 °C for 100 hours after different purifications were performed on DC salt following the procedures outlined in the Experimental section. The standard deviation reported in Fig. 4 was based on at least three coupons for each salt treatment (*i.e.*, non-treated, thermal, and thermal + chemical). Positive corrosion rate is defined as mass loss. Experimental corrosion tests were attempted on non-treated DC, but no results were obtained successfully because the extremely high corrosivity of the non-treated DC caused substantial damage of the experimental setup, *e.g.*, leakage of the furnace vessel. Hence, we did not believe that the results could be used. Instead, the corrosion rate in non-treated DC salt could only be estimated based on the fact that the maximum amount of MgOHCl that the non-treated DC can generate (*i.e.*, 8–9 wt% after 400 °C in Fig. 3) is more than 3 times that for the thermally purified DC (*i.e.*, ∼2.5 wt% after 600 °C in Fig. 3). If we assume that corrosion is roughly linearly correlated to the amount of MgOHCl, then, for first approximation, it is estimated that non-treated DC will produce a corrosion rate of 3200 μm per year or above. It should be noted that corrosion rates based on conversion from mass loss instead of direct measurement of corrosion thickness is used here because (1) intergranular corrosion is commonly seen in metals corroded by molten chlorides which makes it difficult to define such thickness, and (2) the high corrosion rates of “Thermal” in Fig. 4 should not support a build-up layer of corrosion products based on an unpublished study on similar molten chlorides by the same authors. However, for low corrosion rates such as the one reported for “Thermal + Chemical”, it should be cautious to solely rely on mass losses to determine corrosion rates. We acknowledge that there can be errors associated with the growth of corrosion products at the corrosion interface. Our best estimated error for the “Thermal + Chemical” is up to 40 μm per year based on the most likely type of corrosion product (*e.g.*, MgO) and its thickness (*e.g.*, less than 5 μm).

### Salt chemistry analysis

Inductively coupled plasma (ICP)-mass spectroscopy (MS)/optical emission spectroscopy (OES) was used to measure the elemental composition of the cationic impurities present in the non-treated, thermally purified, and chemically purified DC salts (Table 3). Fe, Cr, Mn, and Ni were found to be the major cationic species, in addition to the major salt components of Mg, K, and Na (see Table 1). The increase of Fe, Cr, Mn, and Ni (*i.e.*, major metallic components of stainless steel) after thermal purification is attributed to contamination from the stainless-steel furnace test vessel because corrosion products between salt vapor/HCl vapor and furnace test vessel—especially those formed on the test vessel cap directly above the purification crucible—could drop back into the salt, thus causing contamination. To clarify, salt purification did not use the same setup as the corrosion setup (shown in Fig. 2). An open crucible without a cover was used. Thus, contamination could have occurred and contributed to the increase of Fe, Cr, Mn, and Ni.

**Table 3:** ICP-MS/OES results on non-treated, thermally purified, and thermally/chemically purified DC salts

Salt Treatment	Fe ppm	Cr ppm	Mn ppm	Ni ppm
Non-treated	31	17	3	1
After thermal	7018	327	166	1112
After thermal/chemical	394	120	39	8

## Discussion

The decrease of MgOHCl content from ∼5 wt% in non-treated salt to ∼2.5 wt% in thermally purified salt and to <0.5 wt% in both thermally and chemically purified salt, as shown in Fig. 3, agrees with the decrease of corrosion rate in the corresponding salt, as shown in Fig. 4. This suggests that MgOHCl can be used as a good indicator of the corrosiveness of the molten salt. It has been known from literature^16,18–20^ that intergranular attack of Cr by MgOH^+^ is a common corrosion mechanism in heavily corroded Ni–Fe–Cr alloys such as in the case of the non-treated salt (estimated at >3200 μm per year) and thermally purified salt (∼950 μm per year). When an active metal such as Mg is added into the molten chloride, intergranular corrosion can be significantly reduced, as shown by Ding *et al.*,^21^ who investigated corrosion of Hastelloy C-276 (C276) at 700 °C in a ternary MgCl_2_–KCl–NaCl (60–20–20 mol%) made from commercial NaCl, KCl, and MgCl_2_ with 1 wt% of elemental Mg addition. The corrosion rate of the C276 alloy in Ding *et al.* (29.8 ± 8.7 μm per year) is very close to that of the Haynes 230 corroded in the thermally and chemically purified salt (at 800 °C) in this work. Given the similarity in salt condition and final corrosion rate, it is expected that intergranular corrosion has also been effectively controlled in this work.

Therefore, for Gen3 CSP that aims to use MgCl_2_-containing molten chlorides as HTF and TES to reach a desired 30 year lifespan, it is critical to purify the molten chlorides to minimize MgOHCl content as well as the detrimental intergranular corrosion.

### Effect of thermal purification

Stepwise heating of DC salt at the specified isothermal temperatures is proven to be effective at purifying the salt given the drop of MgOHCl content from a maximum of 8–9 wt% in non-treated DC to ∼2.5 wt% in thermally purified DC (*i.e.*, after 600 °C), as shown in Fig. 3. (Note that the initial MgOHCl was ∼5 wt% in non-treated DC; but the increase of MgOHCl due to hydrolysis of MgCl_2_ during heat treatment must also be taken into account.) This 3- to 4-fold drop in MgOHCl content leads our hypothesis that a significant reduction in corrosion should occur—by comparing the estimated corrosion of non-treated salt to that of the thermally purified salt.

The variation of MgOHCl content during thermal purification also qualitatively corroborates the literature understanding of the chemical changes occurring in hydrated MgCl_2_ system. Hydrolysis of anhydrous MgCl_2_ or MgCl_2_·2H_2_O to form MgOHCl can occur in the temperature range of 210–445 °C.^8,10^ Our measured MgOHCl content indeed shows two increases at the isothermal temperature of 240 °C and 400 °C, which suggest that some MgCl_2_ molecules in DC salt are in the form of MgCl_2_·2H_2_O and MgCl_2_·H_2_O because 240 °C and 400 °C are the corresponding dehydration temperatures, respectively. The variation among non-treated DC at 117 °C and 180 °C may be attributed to inherent inconsistency of salt samples considering the magnitude of the available error bars. The decrease of MgOHCl at 600 °C is the result of MgOHCl thermal decomposition, which is shown to occur at a temperature as low as 415 °C.^8,10^ The thermodynamically predicted onset temperature for MgOHCl thermal decomposition is 555 °C by Kipouros and Sadoway^4^ and 568 °C as calculated by FactSage.^22^

### Effect of metallic Mg during chemical purification

**Fig. 5:**
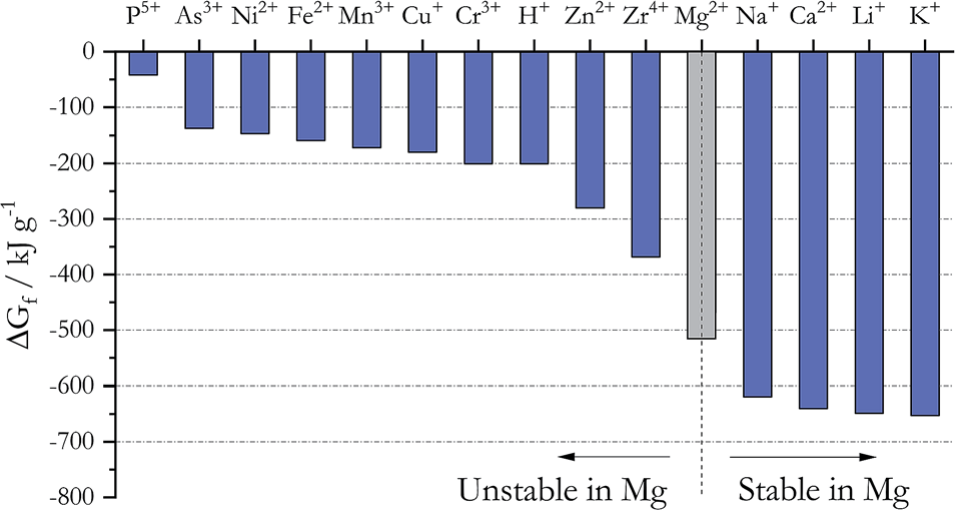
Gibbs free energy of formation for different chlorides at 827 °C. The cations to the left of Mg^2+^ are unstable in the presence of elemental Mg whereas the cations to the right of Mg^2+^ are stable.

Addition of active metals such as Mg or Zr can reduce MgOHCl, as proposed by SRNL. The Ellingham diagram in chloride systems^23^ (Fig. 5) gives the Gibbs free energy of formation Δ*G*_f_ for different cations, which predicts the following: to remove the major metallic impurities such as Fe cations in the DC salt, one needs to use a metal that forms a more stable cation in the chloride system (*i.e.*, with a more negative Δ*G*_f_). At the same time, the metal cation cannot be more stable than the major constituents of the salt (*i.e.*, Na^+^, K^+^, and Mg^2+^); otherwise, we will lose these salt components (*i.e.*, metallic Ca and Li should not be used). Fig. 5 then predicts that Zn, Zr, and Mg are good candidates for this purpose from the thermodynamic perspective. Mg was selected over Zn and Zr because it does not introduce other elements into the ternary MgCl_2_–KCl–NaCl salt system. As shown in Table 3, the concentrations of Fe, Cr, Mn, and Ni decrease significantly in the thermally/chemically purified salt compared to thermally purified salt. Note that Fe, Cr, Mn, and Ni cations are all less thermodynamically stable than Mg cation, as shown in Fig. 5. Although the absolute magnitude of Fe, Cr, Mn, and Ni after thermal/chemical purification was not as low as expected (primarily due to contamination, as explained in the Experimental section), these substantial decreases indeed prove that elemental Mg is able to remove these cations from the salt. A follow-up study (publication in preparation) showed that the concentrations of Fe, Cr, Mn, and Ni can be reduced to less than 10 ppm. In a separate purification experiment using an alternative chloride salt with Ca and Li impurities, chemical purification with Mg is not able to reduce their content, which also agrees with thermodynamic prediction.

### Metallic Mg as corrosion potential control

Metallic Mg is known to provide cathodic protection to metal alloys in molten chlorides where Mg serves as a sacrificial anode.^16,17,19^ It is generally accepted that Cr leaching is the major corrosion mechanism of Cr-containing alloys in molten chlorides.^16,17,19,20,24^ Past work from SRNL^16,17^ showed that without Mg, the corrosion potential of a Fe–Ni–Cr alloy is higher than that of CrCl_2_ such that Cr in the alloy will leach and form CrCl_2_. Addition of Mg shifts the corrosion potential of the alloy by more than 0.5 V to be lower than that of both CrCl_2_ and CrCl_3_—meaning that Cr in the alloy is thermodynamically stabilized. Also, adding elemental Mg into molten chlorides during corrosion testing also significantly reduces the corrosion current—by two orders of magnitude—of the reaction

6 Cr + 2Cl^−^ → CrCl_2_ + 2e^−^

which represents the major mechanism of Cr corrosion in Cr-containing alloys. In this case, the oxidation reaction of metallic magnesium

7 Mg → Mg^2+^ + 2e^−^

provides the majority of the anodic current (rather than that provided by the reaction given by eqn (6)), which balances the cathodic current provided by the following reaction:

8 CrCl_3_ + e^−^ → CrCl_2_ + Cl^−^

The anodic current of reaction in eqn (6), which is directly related to the corrosion of Cr in metal alloys, is lowered; so, addition of elemental Mg should reduce Cr leaching.

### Metallic Mg to facilitate oxygen scavenging

In addition to its use as potential control of corrosion, elemental Mg has a second use to promote oxygen scavenging. Based on eqn (4b), elemental Mg can reduce MgOH^+^ and form MgO and H_2_:

9 2MgOH^+^ + Mg = Mg^2+^ + 2MgO + H_2_

The fundamental cause of MgOH^+^ (or MgOHCl) formation is the reaction of MgCl_2_ with water (and oxygen) in the chloride salt as shown by eqn (2a) and (2b). These soluble MgOH^+^ ions then carry the oxygen atoms from water and become reactive with components in the alloy such as Cr, Mn, and Fe (eqn (4b)). After purification, MgO carries over the oxygen from MgOH^+^ as one of the purification products. The low solubility of MgO in molten chloride (<0.2 wt%^25,26^) suggests that these oxygen atoms, once carried by MgO molecules, should be a minor source of corrosiveness. Eqn (9) then suggests that elemental Mg promotes the reaction of oxygen scavenging during the initial salt purification stage by transforming the most detrimental oxygen-containing species in the molten salt, *i.e.*, soluble MgOH^+^, to MgO. It also suggests that the presence of Mg in the molten salt is important for continued protection of the metal alloys against corrosion as oxygen and moisture ingress (*e.g.*, through flanges and valves) can form more MgOH^+^, which is expected during normal CSP plant operation.

**Fig. 6:**
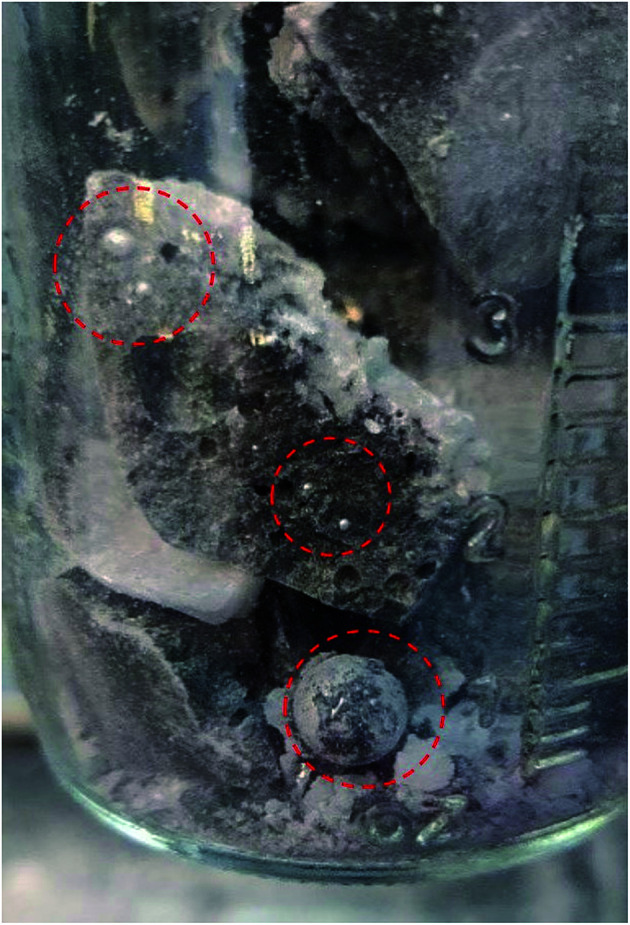
Photo showing the dark sludge phase formed at the bottom of the salt after chemical purification of Mg. The excess Mg droplets are circled in red.

**Fig. 7:**
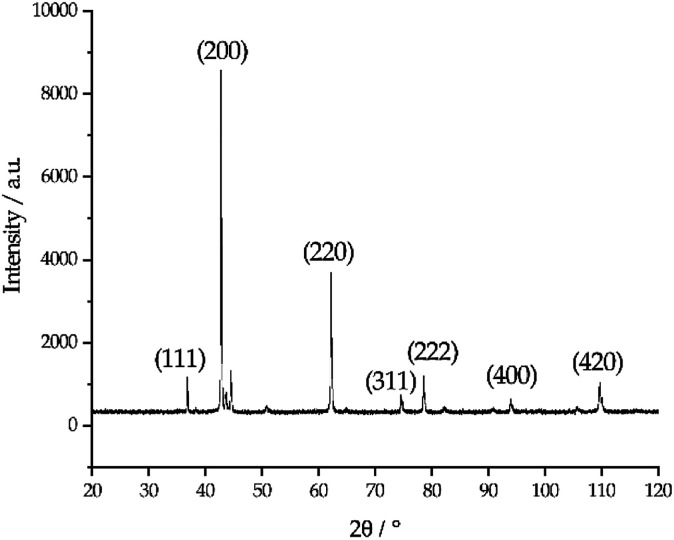
X-ray diffraction pattern of the collected particles in the dark sludge phase. The numbers in the parentheses indicate the crystallographic orientations of MgO reference corresponding to each major diffraction peaks.

The photo in Fig. 6 shows a dark sludge phase formed at the bottom of the chloride salt after chemical purification with Mg. The sludge was dissolved in DI water to remove residual chloride salt and filtered through a 450 nm filter paper. The collected particles were analyzed by X-ray diffraction. Fig. 7 shows that the collected particles in the sludge phase were identified as predominantly MgO. The main diffraction peaks at 2*θ* values of 42.8°, 62.2°, 78.5°, 36.8°, 109.6°, 74.6°, and 93.9° are attributed to the MgO's crystal orientations of (200), (220), (222), (111), (420), (311) and (400), respectively (JCPDS card no. 45-0946 and known literature^27–29^). Only two peaks around 2*θ* of 43–45° and one minor peak around 2*θ* of 51° cannot be attributed to MgO. The XRD result confirms the effectiveness of Mg to promote oxygen scavenging as shown in eqn (9) because reduction of MgOHCl by Mg is the major chemical pathway to form a significant amount of MgO particles. Thermal decomposition of MgOHCl to form MgO (eqn (3)) alone is not able to produce such a large quantity of MgO because the amount of sludge produced after thermal purification was found to be smaller. MgO formation also indicates that Mg is a consumable during the purification process. Therefore, in the future, we need to investigate how much and how frequently Mg should be replenished if oxygen and moisture ingress in the CSP plants is inevitable.

### Amount of Mg added during chemical purification

1.7 wt% of metallic Mg used during chemical purification (the specific amount was provided by SRNL to NREL) is a conservative amount to ensure that Mg is in absolute excess during purification and that most MgOHCl can be reduced to minimize the corrosiveness of the salt during corrosion testing. No optimization of the Mg amount has been performed. The analytical titration method measured that the MgOHCl content was around 2–3 wt% after thermal purification and before chemical purification (see Fig. 3). The stoichiometry in eqn (9) predicts that for 2–3 wt% of MgOHCl, roughly 0.31–0.47 wt% of Mg should be used to ensure complete reduction of MgOHCl. Therefore, an excess of Mg is expected if using 1.7 wt% of Mg. Fig. 6 also proves that 1.7 wt% of Mg is in excess because droplets of Mg are clearly observed in the sludge phase. Therefore, Mg addition needs to be optimized in terms of amount, form factor, and temperature at which addition should occur. This optimization will minimize materials and processing costs because Mg is significantly more expensive than the salt at >$4000/MT. For CSP to be cost competitive, it is essential to minimize the amount of Mg used during initial purification and/or plant operation.

## Conclusions

This work confirmed previous literature understanding of the complex chemistry of MgCl_2_-containing salts and its dehydration strategy. We leveraged such knowledge and demonstrated experimentally that the corrosion rates of Haynes 230 in a MgCl_2_–KCl–NaCl ternary chloride salt can be significantly reduced from >3200 μm per year (estimated) to about 40 μm per year by (1) a thermal purification process as well as (2) a chemical purification process with Mg as corrosion potential control and oxygen scavenger. As shown by the correlation between MgOHCl content in the salt and corrosion rate at different stages of purification, MgOHCl, or soluble MgOH^+^, is primarily responsible for corrosion. Therefore, the corrosion mitigation method using the thermal and chemical purification process is effective because it reduces the MgOHCl content by an order of magnitude to prevent corrosion due to formation of HCl gas and soluble MgOH^+^ ions.

In the future, we plan to optimize the purification process, which aims at (1) reducing the total amount of elemental Mg added to lower the cost at industrial scale and (2) combining the thermal and chemical purification processes to reduce overall energy use.

## Conflicts of interest

There are no conflicts of interest to declare.

## Supplementary Material

## References

[cit1] Guan H. and Wu H., in 2011 International Conference on Electric Technology and Civil Engineering (ICETCE), 2011, pp. 5666–5669

[cit2] Savinkova E. I., Lelekova R. P. (1978). J. Appl. Chem. USSR.

[cit3] Galwey A. K., Laverty G. M. (1989). Thermochim. Acta.

[cit4] Kipouros G. J., Sadoway D. R. (2001). J. Light Met..

[cit5] Kashani-Nejad S., Ng K. W., Harris R. (2004). Metall. Mater. Trans. B.

[cit6] Kashani-Nejad S. , Oxides in the Dehydration of Magnesium Chloride Hexahydrate, PhD thesis, Department of Mining, Metals and Materials Engineering, McGill University, 2005

[cit7] Huang Q.-Z., Lu G.-M., Wang J., Yu J.-G. (2010). Metall. Mater. Trans. B.

[cit8] Eom H.-C., Park H., Yoon H.-S. (2010). Adv. Powder Technol..

[cit9] de Bakker J., Peacey J., Davis B. (2012). Can. Metall. Q..

[cit10] Huang Q., Lu G., Wang J., Yu J. (2011). J. Anal. Appl. Pyrolysis.

[cit11] Rammelberg H. U., Schmidt T., Ruck W. (2012). Energy Procedia.

[cit12] Kashani-Nejad S., Ng K. W., Harris R. (2005). Metall. Mater. Trans. B.

[cit13] Ding W., Bonk A., Gussone J., Bauer T. (2018). Journal of Energy Storage.

[cit14] Maksoud L. and Bauer T., in 10th International Conference on Molten Salt Chemistry and Technology, Shenyang, China, 2015

[cit15] Klammer N., Engtrakul C., Zhao Y., Wu Y., Vidal J. A Novel Method to Determine MgO And MgOHCl in Chloride Molten Salts. Anal. Chem..

[cit16] Garcia-Diaz B. L., Olson L., Martinez-Rodriguez M., Fuentes R., Colon-Mercado H., Gray J. (2016). J. SC. Acad. Sci..

[cit17] Mehrabadi B. A. T., Weidner J. W., Garcia-Diaz B., Martinez-Rodriguez M., Olson L., Shimpalee S. (2017). J. Electrochem. Soc..

[cit18] Ding W., Bonk A., Bauer T. (2018). Front. Chem. Sci. Eng..

[cit19] Mehrabadi B., Weidner J. W., Garcia-Diaz B., Martinez-Rodriguez M., Olson L., Shimpalee S. (2016). J. Electrochem. Soc..

[cit20] Sun H., Wang J., Li Z., Zhang P., Su X. (2017). Sol. Energy.

[cit21] Ding W., Shi H., Jianu A., Xiu Y., Bonk A., Weisenburger A., Bauer T. (2019). Sol. Energy Mater. Sol. Cells.

[cit22] Bale C. W., Bélisle E., Chartrand P., Decterov S. A., Eriksson G., Gheribi A. E., Hack K., Jung I., Kang Y., Melançon J., Pelton A. D., Petersen S., Robelin C., Sangster J., Spencer P., Van Ende M. (2016). Calphad.

[cit23] Reed T. B. , Free Energy of Formation of Binary Compounds, MIT Press, Cambridge, MA, 1971

[cit24] Cho H., Van Zee J. W., Shimpalee S., Tavakoli B. A., Weidner J. W., Garcia-diaz B. L., Martinez-rodriguez M. J., Olson L., Gray J. (2016). Corrosion.

[cit25] Ito M., Morita K. (2004). Mater. Trans..

[cit26] Martin R. L., West J. B. (1962). J. Inorg. Nucl. Chem..

[cit27] Dercz G., Prusik K., Pajak L., Pielaszek R., Malinowski J. J., Pudlo W. (2009). Mater. Sci..

[cit28] Maurya A., Bhatia N. (2017). Int. J. Eng. Res. Dev..

[cit29] Wang B., Xiong X., Ren H., Zhiyu H. (2017). RSC Adv..

